# Hepatitis C and HIV incidence and harm reduction program use in a conflict setting: an observational cohort of injecting drug users in Kabul, Afghanistan

**DOI:** 10.1186/s12954-015-0056-z

**Published:** 2015-10-16

**Authors:** Catherine S. Todd, Abdul Nasir, Mohammad Raza Stanekzai, Katja Fiekert, Heather L. Sipsma, David Vlahov, Steffanie A. Strathdee

**Affiliations:** Department of Obstetrics & Gynecology, College of Physicians and Surgeons, and Heilbrunn Department of Population & Family Health, Columbia University, Mailman School of Public Health, PH 16-69, 622 West 168th Street, New York, NY 10032 USA; Health Protection and Research Organisation, Street 4, Taimani, Kabul, Afghanistan; Department of Epidemiology, Yale School of Public Health, 60 College Street, P.O. Box 208034, New Haven, CT 06520-8034 USA; Department of Community Health Systems, University of California, San Francisco School of Nursing, 2 Koret Way, #N-319X UCSF Box 0602, San Francisco, CA 94143-0602 USA; Division of Global Public Health, University of California San Diego School of Medicine, 9500 Gilman Drive, MC 0507, La Jolla, CA 92093-0507 USA; Asia Pacific Business Unit and Clinical Sciences Division, FHI 360, Sindhorn Building, 130-132 Wittayu Road, Bangkok, 10330 Thailand

**Keywords:** Injecting drug use, Armed conflict, Afghanistan, Hepatitis C virus, HIV, Harm reduction

## Abstract

**Background:**

Armed conflict may increase the risk of HIV and other pathogens among injecting drug users (IDUs); however, there are few prospective studies. This study aimed to measure incidence and potential predictors, including environmental events and needle and syringe distribution and collection program (NSP) use, of hepatitis C virus (HCV) and HIV among IDUs in Kabul, Afghanistan.

**Methods:**

Consenting adult IDUs completed interviews quarterly in year 1 and semi-annually in year 2 and HCV and HIV antibody testing semi-annually through the cohort period (November 2007–December 2009). Interviews detailed injecting and sexual risk behaviors, NSP service use, and conflict-associated displacement. Quarters with peak conflict or local displacement were identified based on literature review, and key events, including insurgent attacks and deaths, were reported with simple counts. Incidence and predictors of HCV and HIV were measured with Cox proportional hazards models.

**Results:**

Of 483 IDUs enrolled, 385 completed one or more follow-up visits (483.8 person-years (p-y)). All participants were male with a median age of 28 years and a median duration of injecting of 2 years. Reported NSP use among the participants ranged from 59.9 to 70.5 % in the first year and was 48.4 and 55.4 % at 18 and 24 months, respectively. There were 41 confirmed deaths, with a crude death rate of 93.4/1000 p-y (95 % confidence interval (CI) 67.9–125) and overdose as the most common cause. HCV and HIV incidence were 35.6/100 p-y (95 % CI 28.3–44.6) and 1.5/100 p-y (95 % CI 0.6–3.3), respectively. Changing from injecting to smoking was protective for HCV acquisition (adjusted hazard ratio (AHR) = 0.53, 95 % CI 0.31–0.92), while duration of injecting (AHR = 1.09, 95 % CI 1.01–1.18/year) and sharing syringes (AHR = 10.09, 95 % CI 1.01–100.3) independently predicted HIV infection.

**Conclusion:**

There is high HCV incidence and high numbers of reported deaths among male Kabul IDUs despite relatively consistent levels of harm reduction program use; peak violence periods did not independently predict HCV and HIV risk. Programming should increase awareness of HCV transmission and overdose risks, prepare clients for harm reduction needs during conflict or other causes of displacement, and continue efforts to engage community and police force support.

## Introduction

The effects of armed conflict and displacement on sexual and blood-borne pathogen infection are complex, with multiple and potentially variable outcomes [1, 2]. Using HIV as an example, systematic reviews of countries where HIV is predominantly sexually transmitted indicate that prevalence among communities of individuals fleeing armed conflict is largely equivalent to or lower than surrounding native communities and that population-level HIV prevalence is generally lower in countries affected by wide-scale armed conflict [3, 4]. However, there is substantial variability in HIV prevalence trends between studies conducted in refugee settlements, and some data suggest sexual violence is associated with HIV infection among displaced women in specific contexts [3, 5]. Comparatively, there is little information regarding impact of conflict in settings where HIV or other pathogens like hepatitis C virus (HCV) transmission is predominantly through injecting drug use [6, 7]. The existing evidence base is considered weak due to lack of comparison populations in assessing levels of risk behavior; limited analysis of individual, network, or environmental factors; and lack of interventions tailored to conflict settings [6].

An illustrative case is Afghanistan, where security has deteriorated since 2006 and injecting as the route of drug administration has become normative in the recent years [8–10]. Key events shaping this context include poor governance and increasing insecurity resulting in conditions favoring unchecked opiate production and, potentially, consumption [11, 12]. Resultant insecurity has increased internal displacement, generally to urban areas with infrastructures already overtaxed by returning refugees, some of whom were drug users [13]. Thus, the Kabul context from 2007 to 2009, the time period during which this observational injecting drug user (IDU) cohort was conducted, was impacted by overcrowding, limited social services, high unemployment, and increasing insurgent attacks, leading to frayed family support structures in an environment with inexpensive, widely available opiates [8, 14].

Within this context, concentrated HCV and HIV epidemics among IDUs have been reported in several Afghan cities [8, 15–17]. However, these assessments cannot infer causality for infection or measure contextual changes related to conflict, harm reduction program use, and HCV or HIV risk [18, 19]. Harm reduction and drug dependency treatment services increased in number and scope during the same period and may have offset risk of HCV or HIV [20, 21]. This study aims to estimate HCV and HIV incidence and modifying factors, including civil insecurity and harm reduction programming, among IDUs in Kabul, Afghanistan.

## Methods

### Setting

Kabul, the Afghan capital, contained 84.1 % of 1350 IDUs enumerated in a 2006 mapping survey [22]. During the study period of June 2007 through December 2009, harm reduction programming in Kabul expanded by both number of programs and range of provided services; by the end of 2009, programs routinely provided drop-in centers, motivational counseling, washing facilities, medical care, and testing for HIV, HCV, and other infections. Some programs offered field outreach activities (generally needle and syringe distribution and collection programs (NSPs)), meals, and night shelter [21]. At the initiation of the study, three harm reduction programs and two no-cost and multiple private for-profit drug dependence treatment programs were operating in Kabul. At the end of the study, there were five harm reduction programs operating, of which four had NSPs. Harm reduction programs had only recently started field-based naloxone distribution in the last year of the study, while distribution of unused injecting supplies (e.g., alcohol swab, gauze, and cooker) in addition to syringes did not occur until after study completion. Opioid substitution therapy was initiated in the last year of the study as one pilot program in Kabul; expansion efforts remain fraught [14]. Insurgent attacks increased sharply in Kabul throughout the study period (95 in 2006 to 175 in 2009) [23].

### Participant eligibility

Participants were enrolled between June 2007 and March 2009 with follow-up through December 2009. Eligibility was limited to IDUs who were aged ≥18 years; reported injecting drugs within the prior 30 days; residing in Kabul; Dari or Pashto speakers; and able to provide informed consent. The protocol was approved by the institutional review boards of the Afghan Ministry of Public Health, Columbia University and the University of California San Diego.

### Measures

Primary outcome measures for this analysis were incident HCV and HIV infection. HCV seroconversion was defined as having newly reactive HCV antibody (HCV Ab) by a rapid diagnostic test (RDT) confirmed by either detectable HCV viremia or HCV Ab. HIV seroconversion was defined as confirmed HIV-1 antibody.

Baseline variables included in this analysis were sociodemographic indicators and variables regarding drug use history and conflict-related events (e.g., age and country of initiating injecting, initiating injecting while a refugee). Interval data analyzed included arrests, incarceration, employment, and homeless status. Interval drug use behaviors included were frequency and duration of injecting, changing from injecting to smoking, sharing syringes or injecting works (e.g., filters, spoons), *khoon bozee* (booting), syringe re-use, and perceived difficulty obtaining clean syringes. Interval NSP use was measured in three ways: any use during the prior 3 months, current use, and discontinuing NSP use. Sexual risk behaviors queried were sex with female sex workers, sex with other males, and any condom use. Other interval events queried included drug dependence treatment, symptoms suggestive of sexually transmitted infection (e.g., dysuria, penile discharge, and/or genital ulcers or warts), and travel outside Afghanistan during the follow-up period.

Variables representing peak conflict periods (the two quarters with the highest number of insurgent attacks) and the quarters surrounding former Russian Cultural Center (RCC) closure (February–May 2009) were generated for analysis. Conflict was quantified by enumerating anti-government attacks in Kabul Province by quarter [23]. For a comparison, an event with displacement resulting not from violence or conflict but from an organized administrative action was also analyzed. The RCC, known as *khana-e-elm-o-farhang*, was a dilapidated building in West Kabul that informally housed between 200–300 drug users and displaced persons until early 2009, when police closed the site with resultant drug user displacement. This closure was pre-announced to occupants and enforced by police patrols without reported excess use of force. Serious events (e.g., deaths, police action) reported by the participants were recorded in field notes and verified where possible. Deaths were generally reported spontaneously by fellow participants, other drug users, or harm reduction workers, though study staff would inquire about the disposition of people not seen recently among members of their social network, which occasionally elicited a reported demise. Following an initial report, a second confirmatory report was required to ensure the correct person was identified as a death case and to obtain the maximum amount of information about cause of death. Cause of death was routinely queried on report but some deaths were reported with no clear cause or the attributed cause could not be verified by a second report. Only death data confirmed by two separate reports are presented.

### Data collection and laboratory methods

Data collection has been previously described in an analysis of baseline data [24]. Briefly, the participants were recruited consecutively from areas of known drug user congregation and through harm reduction programs on scheduled days loosely apportioned by number of IDUs present at those locations, in a variant of time-location sampling. Recruitment sites were initially determined through formative work and through harm reduction programs [25], with locations adjusted monthly based on information from the participants, the harm reduction field workers, and pharmacists of new areas of congregation. There was no set sample size for recruitment at a given site. Sites were distributed throughout Kabul city, with most sites in the western section of the city, consistent with reported and confirmed IDU presence throughout the study period.

For follow-up visits, the participants were met in the field by the study staff and invited to the study office or collaborating harm reduction center for private interview. The study staff comprised former harm reduction workers known to the drug user community and medical professionals (one physician, one lab technician, and one counselor) with experience working with drug users. The staff members were trained in human subject research, interview techniques, and voluntary counseling and testing and also the shadowed field workers in operating harm reduction programs in Afghanistan and Iran to gain additional familiarity with the drug-using community and understand NSP implementation. The study staff members were responsible for all aspects of participant activity, including recruitment, though collaborating harm reduction programs also referred clients for potential participation without compensation.

The participants were assigned a unique study number not linked to any national identifier at enrollment and provided a laminated number “charm” on a thick yarn necklace for them to keep with them for identification through the study period. The participants also provided their preferred name, often a street nickname, to the study staff, who kept a record linking number and name that was stored in a locked secure location when not in use by the study staff in the field. Many participants interacted with the study staff during weekly field visits to the sites they regularly frequented and were well-known to the study staff. However, there were some participants who were not encountered regularly and who provided a street nickname that changed during the study period, making definitive participant identification challenging. The participants not well-known to the study staff were asked to present their study number to the staff prior to proceeding with follow-up visit activities.

Following definitive identification, the participants completed the interviewer-administered questionnaire, received pre-test counseling, and underwent a whole-blood RDT for antibodies to HCV and HIV-1 (SD Bioline, Standard Diagnostics, Kyonggi-do, South Korea). The participants with reactive RDT results provided intravenous specimens, where possible, for confirmatory testing. HCV was confiemd by presence of viremia, tested with polymerase chain reaction (Amplicor, Roche Diagnostics, Mannheim, Germany) or, in absence of viremia, HCV antibody confirmed by recombinant immunoblot assay (Chiron RIBA 3.0 SIA, Chiron Company, Emeryville, California). HIV seroconversion was confirmed with a positive Western blot test (HIV Blot 2.2, Genelabs, Singapore). The participants completed their visit with post-test counseling. Follow-up visits occurred at 3, 6, 9, 12, 18, and 24 months, with serologic testing every 6 months. Treatment referrals were provided for the participants with confirmed HCV and HIV who received results. The participants received in-kind compensation of meals and transportation to and from study visits and injecting and sexual risk reduction supplies, including disposable syringes, alcohol pads, naloxone, and hepatitis B vaccination (for those with non-reactive baseline hepatitis B surface antibody and antigen RDT results).

### Analysis

Descriptive statistics were generated for participants contributing interval data and compared to those lost to follow-up with Chi-square tests, *t* tests, or exact tests, as appropriate. All longitudinal analysis used time defined by visit date. The key outcome measures, HCV and HIV incidence, were calculated with corresponding binomial (HCV) and Poisson (HIV) confidence intervals. Only individuals seronegative for HCV or HIV antibody at baseline with follow-up serologic testing were included in incidence and risk factor analyses.

Risk factors for each outcome were separately assessed using Cox proportional hazards models. First, each outcome was compared to baseline and time-varying predictors and environmental event variables (e.g., former RCC closure) with bivariate Cox regression analysis. Next, factors associated with each outcome at the *p* ≤ 0.20 level in bivariate analysis were eligible for entry into multivariable models. For models with more than one predictor, likelihood ratio test was used to select the best fitting model. Confounders were defined as variables entering the multivariable model that altered the hazard ratio (HR) in bivariate analysis by >10 % for a statistically significant predictor variable. Final models included predictors from the model with best fit and any identified confounders.

For HCV, a separate analysis to better delineate the impact of peak violence on acquisition was performed, as the second peak violence period occurred within 4 months of study closure, potentially excluding cases resulting from behaviors during that period. A forward-lagged variable for peak violence was created for quarter 7, and the quarter during which cases acquired during the peak violence period (Quarter 5) would have been detectable. Statistical significance was defined as two-sided *p* ≤ 0.05. All analysis was performed with Stata 11.0 (Stata Corporation, College Station, Texas).

## Results

### Participant characteristics and cohort events

Of 483 participants, 386 (79.9 %) completed one or more follow-up visits (438.8 person-years (p-y)). The participants could present for any follow-up visit within a specified time interval, and missing one interval visit did not preclude later eligibility. Follow-up rates were 83.9 % (323/385), 77.9 % (300/385), 69.6 % (265/381), 69.2 % (252/364), 55.6 % (153/275), and 38.7 % (65/168) of those eligible at 3, 6, 9, 12, 18, and 24 months, respectively. The participants lost to follow-up were significantly less likely to have prevalent HCV or have initiated injecting within the last year and more likely to have initiated injecting as a refugee or outside Afghanistan, be homeless, or have injected for a longer duration (Table 1).

**Table 1:** Differences between male injecting drug users retained and lost to follow-up in Kabul, Afghanistan, 2007–2009

	Baseline-only group (*n* = 97)	Cohort group (*n* = 386)	
Variable	Median (IQR)	Median (IQR)	*p* value
Age (years)	27, (24–32)	28 (24–35)	0.10
Duration of injecting (years)	1 (0–4)	2 (1–6)	<0.01
Age initiated injecting (years)	25 (21–28)	24 (21–29)	0.92
Level of education (years)	5 (2–9)	5 (0–8)	0.69
Variable	*n* (%)	*n*, (%)	*p* value
Ever married	51 (53)	173 (45)	0.17
Born in Afghanistan	85 (88)	333 (87)	0.73
Lived outside Afghanistan in the last 5 years	60 (62)	250 (65)	0.51
Homeless at enrollment	9 (9)	99 (26)	<0.01
Employed at enrollment	11 (11)	45 (12)	0.91
Prior incarceration	60 (62)	242 (63)	0.79
Initiated injecting in Afghanistan	76 (78)	245 (64)	<0.01
Initiated injecting as a refugee^a^	18 (20)	124 (36)	<0.01
Initiated injection within the last 12 months	33 (34)	78 (20)	<0.01
Shared needles/syringes within 3 months of enrollment (baseline report)	2 (2)	70 (8	0.06
Shared injecting equipment within 3 months of enrollment (baseline report)	26 (27)	106 (28)	0.90
Ever inject/re-aspirate blood	70 (72)	267 (69)	0.64
NSP service use at enrollment	54 (56)	204 (53)	0.67
Receive prior addiction treatment	10 (10)	60 (16)	0.18
HCV infection at baseline	18 (19)	156 (40)	<0.01
HIV infection at baseline	1 (1)	9 (2)	0.70

*HIV* human immunodeficiency virus, *NSP* needle and syringe distribution and collection program, *IQR* interquartile range, *n* number

^a^Of 437 participants who were ever refugees

**Fig. 1:**
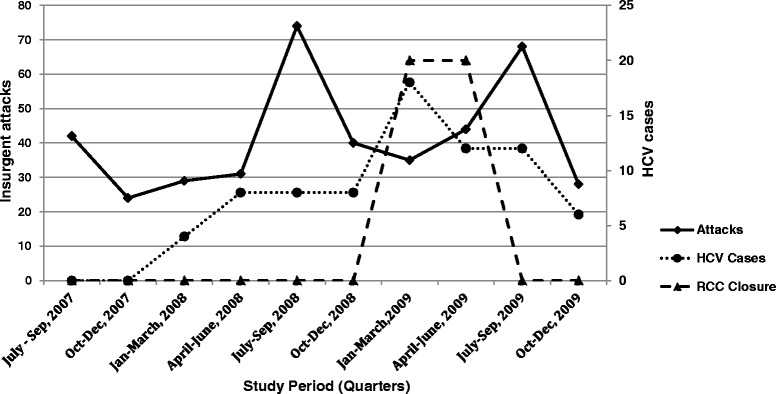
Insurgent attacks and incident hepatitis C cases among male injecting drug users in Kabul, Afghanistan, June 2007–December 2009 (*n* = 191)

HCV incidence, insurgent attacks, and RCC closure during the study period are summarized in Fig. 1. Of 415 participants eligible for hepatitis B vaccination, 95.7, 64.1, and 30.8 % received first, second, and third doses, respectively. Half or more of the participants reported NSP use at any time during the cohort period; NSP use ranged from 59.9 to 70.5 % in the first year of participation and was 48.4 and 55.4 % at 18 and 24 months, respectively. The crude death rate was 93.4/1000 p-y (95 % confidence interval (CI) 67.9–125); of 41 confirmed deaths, 43.9 % (*n* = 18) were reported to result from overdose. Other reported causes of death included exposure during inclement weather or snow (*n* = 6), femoral or other abscess (*n* = 3), trauma (e.g., hit by car, fell down stairs, and beaten by police) (*n* = 3), chest infection (*n* = 2), internal bleeding (*n* = 1), and unknown (*n* = 8).

### HCV and HIV incidence and risk factors

Of 333 participants contributing follow-up data at visits with serologic testing, 191 were at risk for HCV infection and contributed 211 p-y, while 316 were at risk for HIV infection, contributing 391 p-y for analysis. The incidence and median time to infection for HCV were 35.6 cases/100 p-y (95 % CI: 28.4–44.6) and 9.3 months and, for HIV, were 1.5 cases/100 p-y (95 % CI: 0.6–3.3) and 18.3 months.

Risk of HCV acquisition increased with any NSP use, while transitioning from injecting to smoking, sharing injecting works, NSP discontinuation, and, marginally, exposure to peak conflict periods were negatively predictive in bivariate analysis (Table 2). No sexual risk behavior or other conflict-related variables were significant predictors in bivariate analysis (data not shown). To further describe the relationship between conflict and HCV acquisition, a lagged variable restricted to 6 months following the quarter with the greatest number of insurgent attacks was assessed and was not significant (HR = 0.97, 95 % CI 0.56–1.70). In multivariable modeling, only changing from injecting to smoking remained protective for HCV acquisition, reducing risk by 47 % (Table 2). Sharing injecting works confounded this relationship, increasing risk by 6 %.

**Table 2:** Factors predictive of HCV and HIV among male injecting drug users in Kabul, Afghanistan, 2006–2009

Variable	HCV (*n* = 191, 211 p-y)	HCV	HIV (*n* = 316, 391 p-y)	HIV
	HR, (95 % CI)	AHR, (95 % CI)	HR, (95 % CI)	AHR, (95 % CI)
Duration of injecting use			1.08, (1.00–1.17)	1.09, (1.01–1.17)^a^
Sharing of syringes/needles			11.23,	(1.29–98.0) 10.08, (1.01–100.3)^a^
Sharing of injecting supplies	0.56, (0.35–0.90)			
Any NSP use during interval	1.72, (1.07–2.76)			
Interval NSP discontinuation	0.61, (0.38–0.98)			
Changed from injecting to smoking	0.47, (0.29–0.77)	0.53, (0.31–0.92)^b^	0.23, (0.03–1.93)	
Peak quarters of attack exposure^c^	0.65, (0.40–1.04)*			

*AHR* adjusted hazard ratio, *CI* confidence interval, *HCV* hepatitis C virus, *HIV* human immunodeficiency virus, *HR* hazard ratio, *NSP* needle and syringe collection and distribution program, *p-y* person-years

*Marginally significant (*p* = 0.07)

^a^Multivariate analysis controlled for confounder interval NSP use (11.23 to 7.19)

^b^Analysis controlled for cofounder interval sharing injecting works (0.47 to 0.53)

^c^Peak quarters of attack refer to quarters 5 and 9 of the study, the two quarters with the largest number (142/415 total) of insurgent attacks in Kabul province

HIV acquisition significantly increased with duration of injecting and sharing syringes in bivariate and multivariate Cox regression models (Table 2). The model with both predictors indicated HIV risk increased by 9 %/year with duration of injecting (adjusted hazard ratio (AHR) = 1.09, 95 % CI 1.01–1.18) and 16-fold for sharing syringes (AHR = 16.1, 95 % CI 1.68–153.1), as duration of injecting increased risk of HIV secondary to sharing syringes. The multivariate model was then adjusted for interval NSP use, which decreased HIV risk secondary to sharing syringes (HR = 11.23 to AHR = 7.19 in model including sharing syringes and interval NSP use); in the final adjusted model, sharing syringes increased HIV risk 11-fold (Table 2). No confounders were identified for the association between duration of injecting and HIV acquisition. Of six new HIV infections, five occurred in men with prevalent HCV, while the remaining participant was diagnosed with incident HCV and HIV at the 6-month follow-up visit.

## Discussion

The results of this study may contribute to evidence concerning conflict and drug use and may inform programming in Afghanistan and other conflict-affected settings [6–8, 26–29]. Key findings include high HCV incidence whose risk was reduced by half with cessation of injecting, low HIV incidence, high death rates, and relatively high rates of NSP use throughout the cohort period.

HCV incidence was quite high, similar to rates reported among other IDU populations in non-conflict settings [30–35]. Change from injecting to smoking was protective against HCV and may have been prompted by desire to enter a drug dependence treatment program. Spaces for no-cost inpatient treatment programs in Kabul are limited and require 1 to 3 months of daily outpatient counseling with demonstrated transition to smoking preferred [9]. Motivation to enter a highly coveted treatment program may have prevented HCV infection, even in an environment where displacement or violence may have favored transition to or continuation of injecting [27–29]. These findings could also be used for primary prevention programming by discouraging transition from smoking to injecting as a means of preventing HCV acquisition.

Sharing injecting works negatively predicted HCV acquisition in bivariate analysis but slightly increased risk among those transitioning to smoking in the final model, potentially representing an unreported resumption of injecting. This finding requires further inquiry as HCV transmission resulting from the use of contaminated injecting paraphernalia is well-established [36]. We hypothesize that increased knowledge surrounding risks of sharing works without availability of these items through harm reduction programs during the study period may have resulted in under-reporting of ongoing sharing. Further, HCV was not as widely emphasized as a risk of injecting as compared to HIV during the study period. This emphasis may have changed, though it is not reflected in treatment offerings as HIV treatment for IDUs is available through one harm reduction program in Kabul while HCV treatment is not available in the public sector and remains very expensive (personal communication, Dr. H. Mansoor). Underestimation of HCV cases may have occurred through attrition from baseline or differential drop-out as participants at risk for HCV were increasingly lost to follow-up compared to those with prevalent infection.

HIV incidence was comparatively low, in contrast to Pakistan, a country affected by violent attacks, and to predictions for conflict-affected contexts [6, 37]. Low HIV incidence may reflect lower per-act risk due to known lower prevalence among Kabul IDUs [17, 24]. HIV acquisition increased with duration of injecting and with interval sharing of syringes, both established risk factors by international consensus and also associated with prevalent infection in this cohort [24, 37–40]. Recent NSP use reduced HIV risk attributable to syringe sharing, yet significantly increased HCV risk in bivariate analysis. We hypothesize that these disparate results were due to environmental influences. Most new HCV infections were detected in quarters 7–9 (February–September, 2009), representing the time surrounding and immediately following RCC closure. During this period of displacement, interruption of harm reduction services, particularly field-based services like NSP, was more likely, potentially leading to unreported new-onset syringe sharing or other risky injecting behaviors. Further investigation is needed to determine NSP service coverage and reasons for discontinuation in this challenging setting. The negative association between exposure to violence and HCV acquisition in bivariate analysis potentially reflects IDUs secluding themselves alone or within well-established networks wherein new transmission is unlikely, even with potential increase in individual risk behaviors. Risk factors for HIV should be interpreted with caution due to very low incidence.

Deaths were common and under-reporting was likely, though the crude death rate was high compared to that reported for Afghan males aged between 20 and 59 years (10.99/1000 p-y) [41]. Study representatives were informed of approximately 30 additional deaths that could not be confirmed. These data should be interpreted with caution as exact cause of death was subject to the same limitations as enumerating deaths due to lack of a reliable death registry and challenges inherent to tracking health outcomes in this marginalized population. For similar reasons, we did not analyze associations between environmental events and reported date of death. Overdose was the most common reported cause of death. During the cohort period, field-based distribution of naloxone was not routinely provided by NSPs until 2009, though it was through the study. NSPs expanded the range of distributed products during the cohort period to include both naloxone and injecting paraphernalia beyond syringes; impact of this change on overdose and HCV incidence should be assessed.

Several additional limitations must be considered. First, the cohort had low follow-up rates, particularly in the second year. This study was very challenging operationally as approximately 20 % of the participants were homeless and many came to Kabul as a first point of repatriation, potentially then moving to home provinces, making it difficult to find the same individual on a regular basis. The study staff knew many participants only by a street nickname, which could change over time, potentially motivated by fear of police action. Further, particularly following RCC closure, police action allegedly led to repeated scattering of the participants throughout Kabul, making wide sampling efforts and regular follow-up difficult. Migration within and outside Kabul, unconfirmed deaths, and incarceration also likely contributed to high attrition. Efforts to equally distribute participant enrollment between known sites of drug user congregation throughout Kabul were based on numbers that varied widely throughout the cohort period. We attempted to mitigate this effect by regularly consulting the outreach workers and the participants with esteemed standing in their social groups as to where to reach current and potential participants, but congregation sites frequently changed without warning. Thus, there may have been bias toward selecting individuals accessing harm reduction programs and returning to the same congregation sites, with resultant higher reported NSP use rates. Some reporting or recall bias may have been present; to minimize this, the participants were asked about behaviors in the last 3 months and the first year comprised quarterly follow-up. Due to low literacy, questionnaires were administered by study representatives, with possible resultant socially desirable response. Audio computer-assisted self-interview was not feasible in this setting.

In summary, while there are important limitations, data indicate Kabul IDUs are caught in the crossfire of environmental changes fostering high HCV incidence and death rates. Growing instability, displacement, and increasing competition for resources in Kabul and limited progress toward improving key environmental elements indicate need for different approaches to harm reduction in this context. Harm reduction and drug dependence treatment programming should increase emphasis on prevention programming for HCV and overdose and develop and test models for service provision or client preparedness during times of displacement to prevent field-based harm reduction service interruption as well as to work with clients to transition from injecting to smoking and prevent transition to injection among smokers. NSP programs are to be commended for relatively high sustained levels of service provision in this challenging setting and for ongoing educational efforts aimed at aligning policing and community support with harm reduction. These interventions, particularly with police, should continue but adaptability of, expansion of, and improved services within harm reduction programs are also indicated to enable drug users to cope with ongoing conflict in this and similar contexts.
